# EDNRA Gene rs1878406 Polymorphism is Associated With Susceptibility to Large Artery Atherosclerotic Stroke

**DOI:** 10.3389/fgene.2021.783074

**Published:** 2022-01-03

**Authors:** Wan Wei, Xianjun Xuan, Jiahui Zhu, Tianwen Chen, Yudan Fang, Jiao Ding, Danfei Ji, Guoyi Zhou, Bo Tang, Xudong He

**Affiliations:** ^1^ Affiliated Hangzhou First People’s Hospital, Zhejiang University School of Medicine, Hangzhou, China; ^2^ Department of Neurology, Hangzhou Ninth People’s Hospital, Hangzhou, China; ^3^ Fourth Clinical Medical College of Zhejiang Chinese Medical University, Hangzhou, China; ^4^ Sir Run Xuedong Shaw Hospital, Hangzhou, China

**Keywords:** polymorphism, large artery atherosclerosis, genotype, stroke, rs1878406

## Abstract

**Objective:** We performed this study to investigate whether the EDNRA gene rs1878406 C > T polymorphism is associated with risk of large artery atherosclerosis (LAA) stroke in the Chinese Han population.

**Methods:** Genotyping of rs1878406 was performed in 1,112 LAA stroke patients and 1,192 healthy controls. Multivariate logistic regression analyses were applied to assess the effect of the rs1878406 C > T polymorphism on susceptibility to LAA stroke.

**Results:** A significant increase of LAA stroke risk was found in the recessive model (TT vs. CC/TC, OR = 1.74, 95% CI = 1.23–2.48, *p* = 0.002) and co-dominant model (TC vs. CC, OR = 1.06, 95% CI = 0.89–1.27, TT vs. CC, OR = 1.79, 95% CI = 1.25–2.55, *p* = 0.006). However, the interaction between age and genotypes of rs1878406 was not statistically significant, and no significant interactive effect was observed between the rs1878406 C > T polymorphism and sex (*p* > 0.05).

**Conclusion:** The rs1878406 C > T polymorphism is associated with increased risk of LAA stroke in the Chinese Han population.

## Introduction

Stroke has become the second leading cause of death and the third cause of disability in humans. Among them, ischemic stroke accounts for about 80% of all strokes (GBD 2015 DALYs and HALE Collaborators, 2016), and it is a major cause of mortality and disability worldwide (Mendis et al., 2015), loading heavy economic burden especially in low- and middle-income countries (Benjamin et al., 2017). The occurrence and development of stroke are related to endothelial injury, atherosclerosis (Hankey, 2017), and traditional risk factors including age, gender, hypertension, diabetes mellitus, hyperlipidemia, and smoking. Besides traditional risk factors, some specific gene locus polymorphisms have also been confirmed to be associated with LAA stroke (Bellenguez et al., 2012; Malik et al., 2018). In recent years, genome-wide association studies (GWAS) have become an important research method for finding complex disease-related susceptibility genes, and many single-nucleotide polymorphisms (SNPs) related to LAA stroke have been discovered (Traylor et al., 2012). The interaction between gene and environmental factors has also received increasing attention.

Endothelin type A receptor (EDNRA) is a receptor for endothelin-1 (ET-1), a potent vasoconstrictor. *EDNRA* is expressed in vascular smooth muscle cells (Yu et al., 1995). ET-1, encoded by the *EDN1* gene located in chromosome 6p21-24, is a potent vasoconstrictor in the body. ET-1 is expressed in several tissues, including endothelial cells and cardiomyocytes (Brunner et al., 2006). After ET-1 being bounded firmly to EDNRA, it activates calcium channels and phospholipase activation pathway to play a long-lasting vasoconstriction effect (Kampoli et al., 2011). It can also promote the proliferation and migration of vascular smooth muscle cells, increasing the ratio of neointima and media, stimulating the production of cytokines and growth factors, and inducing the formation of extracellular matrix and fibers. Hyperplasia is involved in the occurrence and development of atherosclerosis through many ways (Dai and Dai, 2010; Li et al., 2015a). GWAS have identified that SNP rs1878406 C > T polymorphism in *EDNRA* gene is related to carotid plaque (Bis et al., 2011). A following study also associated rs1878406 with coronary artery disease (Hemerich et al., 2015). Therefore, we performed this study to investigate the association between the rs1878406 C > T polymorphism and LAA stroke risk in the Chinese Han population and test whether age or gender interacts rs1878406 to influence LAA stroke risk.

## Materials and Methods

### Study Subjects

We consecutively recruited first-ever LAA stroke patients between January 2015 and December 2019 from the first hospital of Hangzhou Neurology Department. Ischemic stroke was defined as sudden focal neurological deficits lasting ≥24 h, with evidence of cerebral infarction in clinically relevant areas of the brain confirmed by computed tomography (CT) and/or magnetic resonance imaging (MRI). Patients were enrolled if they (1) were 18 years or older; (2) were of Chinese Han ethnicity; (3) had available blood samples; and (4) diagnosed with LAA stroke according to TOAST classification (Adams et al., 1993). Exclusion criteria included severe liver or kidney dysfunction, hematological diseases, malignancies, and autoimmune disorders. The non-stroke controls were recruited from local inhabitants during the same period and the inclusion criteria were as follows: (1) aged 18 years or older; (2) Chinese Han ethnicity; (3) had regular physical examination; (4) no history of transient ischemic attacks, cerebrovascular, cardiovascular, and atherosclerotic diseases; and (5) no history of severe liver or kidney dysfunction, hematological diseases, malignancies, and autoimmune disorders. The study was approved by the Ethical Review Board of the First Hospital of Hangzhou (Hangzhou, China).

### Data Collection

Baseline characteristics included demographic information (age, gender, height, and weight), medical histories (hypertension, diabetes mellitus, hyperlipidemia, and smoking), and laboratory tests. Smokers were those who had smoked ≥100 cigarettes during their lifetime and currently smoke every day or some days (Loke et al., 2014). Routine laboratory investigations were performed after overnight fasting within 24 h of admission. The definition of hyperlipidemia is serum triglyceride ≥150 mg/dl, low-density lipoprotein cholesterol ≥130 mg/dl, high-density lipoprotein cholesterol ≤40 mg/dl in adult males and ≤50 mg/dl in adult females, any use of lipid-lowering drugs, or any self-reported history of hyperlipidemia (Wang et al., 2015).

### Genotyping

All patients took 5 ml of fasting peripheral venous blood in the early morning and placed it in an *EDTA* anticoagulation tube. After centrifugation, the lower layer of blood cells was removed and stored in a refrigerator at −80°C. Genotyping was conducted by SNPscan technology, supported by the Center for Human Genetics Research, Genesky Biotechnology Co. Ltd. (Shanghai, China). About 5% of the samples were randomly selected and genotyped repeatedly to confirm the genotyping credibility, and the results were 100% consistent.

### Statistical Analyses

Statistical analyses were performed with SPSS Statistics Version 22.0 (IBM, Armonk, NY, United States). Student’s *t*-test was conducted for continuous variable. Categorical data were expressed as frequency and percentage. Categorical variables were compared using Chi-square test. The mean age of LAA stroke onset for three polymorphisms was compared using one-way ANOVA. Univariate and multivariate logistic regression analyses were performed to investigate the association of the rs1878406 C > T polymorphism with risk of LAA stroke. Akaike information criterion (AIC) was calculated for selecting the best model for the SNP. All statistical tests were two-sided and *p* < 0.05 was considered statistically significant.

### *In Silico* Analysis

To provide function annotation for rs1878406 polymorphism in *EDNRA* gene, we performed *in silico* analysis using Haploreg v4.118 (https://pubs.broadinstitute.org/mammals/haploreg/haploreg.php).

## Results

### Baseline Characteristics of the Subjects

A total of 1,112 LAA stroke patients and 1,192 healthy controls were recruited in this study. Baseline clinical and demographic characteristics are shown in Table 1. Compared with the controls, patients were older (*p* < 0.001) and had a higher proportion of male and traditional risk factors for ischemic stroke, such as history of hypertension, diabetes mellitus, hyperlipidemia, and smoking (*p* < 0.001).

**TABLE1:** Baseline clinical and demographic characteristics of the LAA stroke patients and controls.

Characteristic	Case	Control	*p*
N	1,112 (48.26%)	1,192 (51.74%)	
Sex			
Female	290 (26%)	440 (37%)	<0.001
Male	822 (74%)	752 (63%)	
Age	61.40 ± 10.31	57.42 ± 10.16	<0.001
≤60	496 (45%)	810 (68%)	<0.001
>60	616 (55%)	382 (32%)	
Hypertension			
No	292 (26%)	851 (71%)	<0.001
Yes	820 (74%)	341 (29%)	
Diabetes mellitus			
No	740 (67%)	1,041 (87%)	<0.001
Yes	372 (33%)	151 (13%)	
Hyperlipidemia			
No	677 (60.9%)	892 (74.8%)	<0.001
Yes	435 (39.1%)	300 (25.2%)	
Smoker			
No	632 (57%)	823 (69%)	<0.001
Yes	480 (43%)	369 (31%)	

### Association of rs1878406 Polymorphism With Age of Onset of LAA Stroke

**FIGURE 1:**
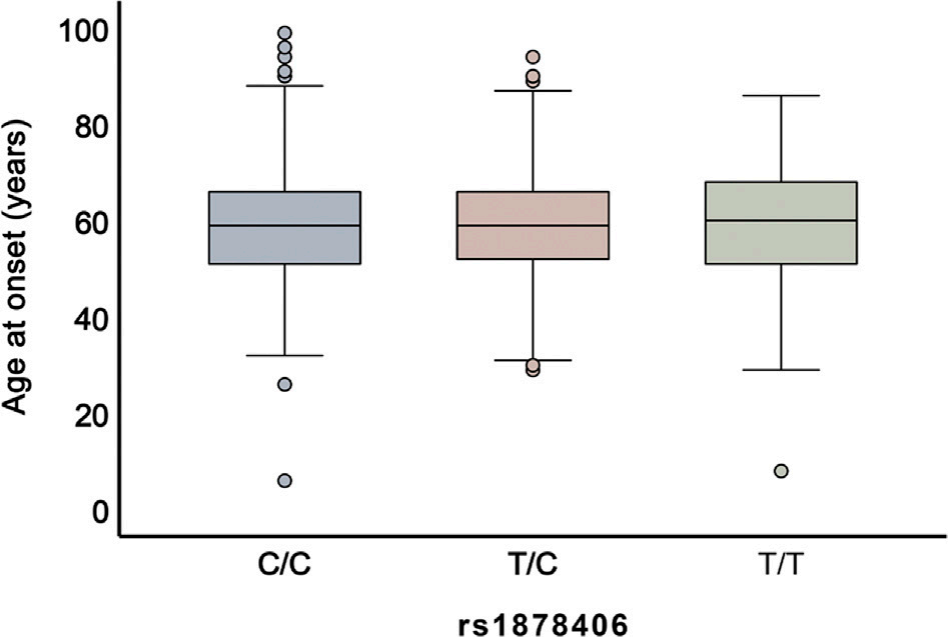
Association between rs1878406 polymorphism and age at onset of LAA stroke. Box plot of age at LAA stroke onset for three different genotypes of patients with rs1878406.

We first evaluated the correlation between the rs1878406 polymorphism and the age of onset of LAA stroke. As shown in Figure 1, these three polymorphisms were not significantly related to the age at onset of LAA stroke (*p* > 0.05).

### Association of rs1878406 C > T Polymorphism With the Risk of LAA Stroke

The frequencies of CC, TC, and TT genotypes were 60.1%, 35.3%, and 4.5%, among the LAA stroke patients, respectively, and 56.8%, 35.5%, and 7.6%, among the controls, respectively. According to the AIC values, the recessive model is the best-fitting model. In the recessive model, compared with CC and TC genotype, TT genotype of the rs1878406 was associated with significantly increased risk of LAA stroke (OR = 1.74, 95% CI = 1.23–2.48, *p* = 0.002; Table 2). After adjustment for gender, age, hypertension, diabetes mellitus, hyperlipidemia, and smoking, the association remained significant (OR = 1.80, 95% CI = 1.19–2.73, *p* = 0.005 for CC/CT vs. TT). The rs1878406 C > T polymorphism also had a common effect on LAA stroke in the co-dominant model (TC vs. CC, OR = 1.06, 95% CI = 0.89–1.27; TT vs. CC, OR = 1.79, 95% CI = 1.25–2.55; *p* = 0.006). However, the risk effect was not statistically significant in the dominant model (*p* = 0.137). A dose–response relationship between the T allele and risk of LAA stroke was determined by the log-additive model (OR = 1.20, 95% CI = 1.03–1.41, *p* = 0.022; Table 2).

**TABLE 2:** Association of the rs1878406 C > T polymorphism with risk of LAA stroke.

Genetic models	Genotype	Case	Control	Crude OR (95%CI)	Crude *p* value	AIC	Adjusted OR (95%CI)	*p* ^a^
Codominant	C/C	717 (60.1%)	632 (56.8%)	1	0.006	3,186.9	1	0.016
	T/C	421 (35.3%)	395 (35.5%)	1.06 (0.89–1.27)			1.07 (0.87–1.32)	
	T/T	54 (4.5%)	85 (7.6%)	1.79 (1.25–2.55)			1.85 (1.21–2.83)	
Dominant	C/C	717 (60.1%)	632 (56.8%)	1	0.106	3192.6	1	0.137
	T/C-T/T	475 (39.9%)	480 (43.2%)	1.15 (0.97–1.35)			1.16 (0.95–1.41)	
Recessive	C/C-T/C	1,138 (95.5%)	1,027 (92.4%)	1	0.002	3,185.4	1	0.005
	T/T	54 (4.5%)	85 (7.6%)	1.74 (1.23–2.48)			1.80 (1.19–2.73)	
Log-additive	—	—	—	1.19 (1.04–1.36)	0.011	3,188.8	1.20 (1.03–1.41)	0.022

a Adjusted for age, blood pressure, diabetes, hypertension, hyperlipidemia, and smoking.

### Subgroup Analysis and Interaction Analysis According to Age and Sex

**FIGURE 2:**
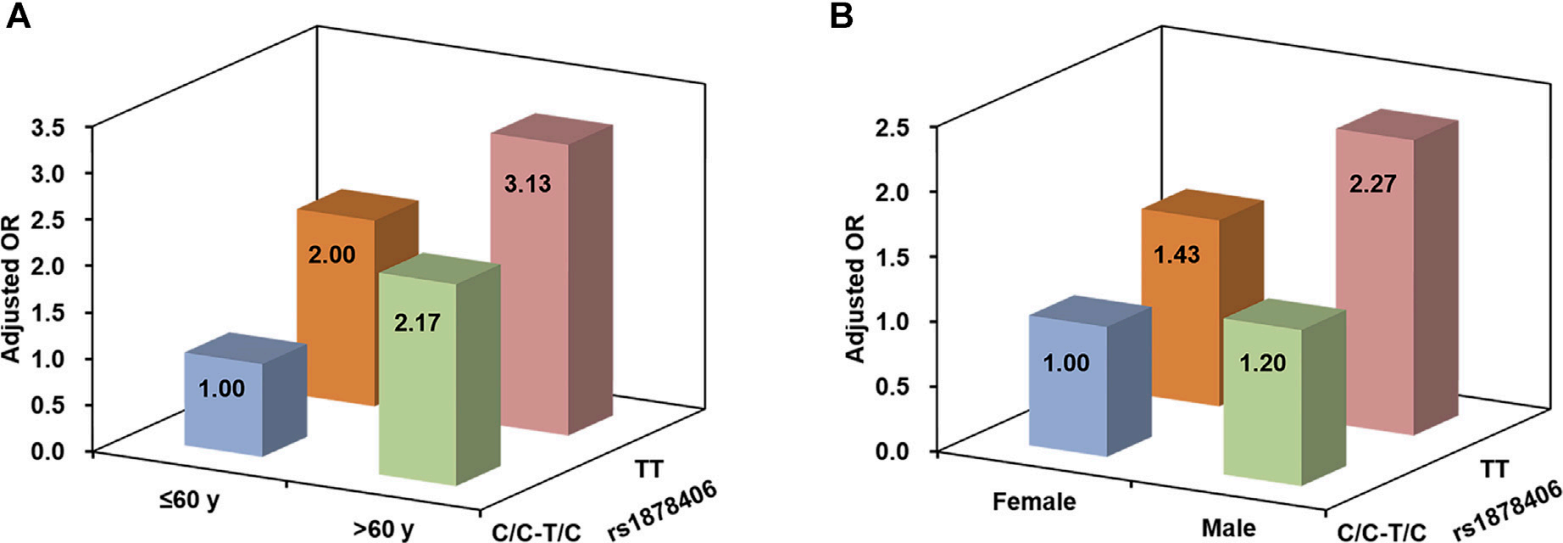
**(A)** Adjusted OR of LAA stroke according to rs1878406 and age; the interaction between rs1878406 and age was non-significant (*p*_interaction_ = 0.442). **(B)** Adjusted OR of LAA stroke according to rs1878406 and sex; the interaction between rs1878406 and sex was non-significant (*p*_interaction_ = 0.553).

Table 3 shows the effect of rs1878406 on the risk of LAA stratified by age. In adults aged ≤60 years, the T allele of rs1878406 showed significant association with risk of LAA stroke (CC/CT vs. TT, OR = 2.00, 95% CI = 1.16–3.45, *p* = 0.013). In adults aged >60 years, however, no significant association was found. In the stratified analysis by sex, the T allele of rs1878406 significantly increased the risk of LAA stroke in males (CC/CT vs. TT, OR = 1.89, 95% CI = 1.14–3.11, *p* = 0.011; Table 4). In females, however, we did not detect any significant association of rs1878406 with risk of LAA stroke. The results suggested potential interactions between rs1878406 polymorphism, age, and sex in the etiology of LAA stroke. However, the interaction between age and genotypes of rs1878406 was not statistically significant (*p*_interaction_ = 0.442; Figure 2A). Similarly, there was no statistical evidence for interaction between these rs1878406 polymorphism and sex on the risk of LAA stroke (*p*_interaction_ = 0.553; Figure 2B).

**TABLE 3:** Age-stratified analysis of the association between the rs1878406 C > T polymorphism and risk of LAA stroke.

Genotypes	≤60				>60			
	Control	Case	OR (95% CI)	*p* ^a^	Control	Case	OR (95% CI)	*p* ^a^
C/C-T/C	775	458	1	0.013	363	569	1	0.248
T/T	35	38	2.00 (1.16–3.45)		19	47	1.44 (0.78–2.66)	

a Adjusted for blood pressure, diabetes, hypertension, hyperlipidemia, and smoking.

**TABLE 4:** Sex-stratified analysis of the association between the rs1878406 C > T polymorphism and risk of LAA stroke.

Genotypes	Female				Male			
	Control	Case	OR (95% CI)	*p* ^a^	Control	Case	OR (95% CI)	*p* ^a^
C/C-T/C	418	270	1	0.308	720	757	1	0.011
T/T	22	20	1.43 (0.68–3.02)		32	65	1.89 (1.14–3.11)	

a Adjusted for age, blood pressure, diabetes, hypertension, hyperlipidemia, and smoking.

### Functional Annotation

Bioinformatics analysis by HaploReg v4.1 indicated that rs1878406 could change the binding affinity of regulatory motifs Myf_4 (Table 5).

**TABLE 5:** Regulatory motifs altered for rs1878406 based on HaploReg v4.1.

PWM ID	PWM match score		C Allele: TTA​TCT​TCA​GTC​TCA​CCT​CCG​AAT​CCT​GTC​AGC​TGT​TAT​CTC​GTA​TTT​AGA​GCT​TAC​CA
	C Allele	T Allele	T Allele: TTA​TCT​TCA​GTC​TCA​CCT​CCG​AAT​CCT​GTT​AGC​TGT​TAT​CTC​GTA​TTT​AGA​GCT​TAC​CA
GATA_known1	12	12.4	HBDHVDYTCTTATCTHYHHWHV
GATA_known4	12.9	12.6	NNHHBSTTATCWHBNDW
Myf_4	13.4	5.9	NDRVCAVCTGYYNNBB

PWM, Position Weight Matrix (Library from Kheradpour and Kellis, 2013).

## Discussion

We confirmed that age, blood pressure, diabetes, hypertension, hyperlipidemia, and smoking were independent risk factors for cerebral infarction by comparing the general data of atherosclerotic cerebral infarction with those of normal controls, which were consistent with the results of previous studies. After adjusting for factors such as gender, age, and hypertension under the recessive model, it was shown that TT genotype at rs1878406 was a risk factor for susceptibility to cerebral infarction as compared with the CC/TC genotype. Under the recessive model, the interaction between age or gender and genotypes of rs1878406 C > T polymorphism was not statistically significant. Altogether, it is found that the T allele at *EDNRA* rs1878406 is a risk factor for cerebral infarction.

The *EDNRA* gene encodes ET-1 receptor A, which plays an important role in effective and durable vasoconstriction (Dai and Dai, 2010; Li et al., 2015a), and is widely distributed in the cardiovascular and central nervous system. Encoded by *EDN1*, ET-1 is a potent vasoconstrictor that is expressed in a variety of tissues, including endothelial cells and cardiomyocytes (Brunner et al., 2006). ET-1 is closely related to cardiovascular and cerebrovascular diseases, and endothelial dysfunction can be found in hypertension, atherosclerosis, diabetes, hyperlipidemia, and cerebrovascular spasm, with increased ET release (Balletshofer et al., 2000). In 1992, Zir et al. (Balletshofer et al., 2000) first confirmed the elevated plasma ET-1 level in patients with acute ischemic cerebral infarction. In recent years, the relationship between ET-1 and ischemic cerebrovascular disease has become a hot topic in basic and clinical medicine. ET-1 mediates endothelial dysfunction mainly by increasing fibroblasts and macrophages. Endothelial damage greatly enhances the sensitivity of blood vessels to ET-1, causing durable contraction of local blood vessels, and ischemia and hypoxia of brain tissue. In addition to its effect on blood vessels, ET-1 can also directly act on nerve cells, causing cell death, accelerating the death of neurons in the hypoxia area by promoting the release of excitatory amino acids (Lin et al., 1990). Studies have shown that ET-1 can bind to its receptor and activate the voltage-sensitive L-type calcium channel, causing extracellular calcium influx and intracellular calcium overload, thus aggravating the damage of nerve cells (Marsden et al., 1989). All the above lines of evidence provide us with the theoretical basis that the rs1878406 variant in *EDNRA* may be related to cerebral infarction.

Multiple previous studies have shown that the rs1878406 variant is associated with atherosclerosis and endothelial dysfunction. For example, in the 2011 meta-analysis by Bis et al. (2011), it was found that the C allele of rs1878406 was associated with lower risk of plaque. However, the T allele was associated with a 22% increased odds of the presence of plaque. These associations may provide important insights into the pathophysiological mechanisms relating the genes to atherosclerosis and subsequent artery disease. Another study (Li et al., 2015b) explored the potential relationship between EDNRA rs1878406 polymorphisms and the carotid intima-media thickness (IMT) levels, but no statistically significant differences were found when this polymorphism was assessed according to carotid IMT values. Instead, they identified a significant interaction of gender with this variant rs1878406 in the *EDNRA* gene. For gene–gender interaction on common carotid arteries IMT, the adjusted mean for men carrying the GA/GG genotype of *EDNRA* SNP rs1878406 was 1.18 times higher than that for men carrying the AA genotype. This may be due to the fact that the sample size of their study was relatively small. Zhang et al. (2017) found in their analysis of the Han population that the rs1878406 TT/TC genotype could be a significant risk factor for severe multiple coronary artery lesions. Moreover, our results indicated that rs1878406 could change the binding affinity of regulatory motifs Myf_4. The above results of previous studies are consistent with our studies, suggesting that the C→T mutation in the *EDNRA* gene rs1878406 may influence the function of ET-1, thereby affecting the occurrence of atherosclerosis.

Our study has some limitations. First of all, we only analyzed the Han population, which is not representative of other ethnic groups. More international institutions should further combine for sample collection, so that possible geographical and ethnic differences can be compared in the future. Secondly, the sample size of this study is relatively small, and further larger sample study is required. In addition, there is a lack of in-depth study on the related mechanism of rs1878406 polymorphism increasing the risk of cerebral infarction, and further functional verification can be conducted, including *in vivo* and *in vitro* experiments.

In conclusion, the results of this study suggest that individuals carrying the *EDNRA* rs1878406 TT genotype may be a risk factor against cerebral infarction. However, to explore the relationship between rs1878406 and pre-cerebral infarction, we need to conduct joint analysis on the basis of the largest sample size as possible, so as to provide a reliable theoretical basis for the early prevention and treatment of cerebral infarction.

## Data Availability

The data presented in the study are deposited in the EMBL-EBI repository, accession number PRJEB48922.
